# Combined Therapy With Avastin, a PAF Receptor Antagonist and a Lipid Mediator Inhibited Glioblastoma Tumor Growth

**DOI:** 10.3389/fphar.2021.746470

**Published:** 2021-09-24

**Authors:** Valerie A. Cruz Flores, Hemant Menghani, Pranab K. Mukherjee, Luis Marrero, Andre Obenaus, Quan Dang, Larissa Khoutorova, Madigan M. Reid, Ludmila Belayev, Nicolas G. Bazan

**Affiliations:** ^1^ Neuroscience Center of Excellence, Louisiana State University Health Sciences Center, New Orleans, LA, United States; ^2^ Department of Pediatrics, Hematology-Oncology, Louisiana State University Health Sciences Center, New Orleans, LA, United States; ^3^ Department of Orthopaedic Surgery, Louisiana State University Health Sciences Center, New Orleans, LA, United States; ^4^ Department of Pediatrics, School of Medicine, University of California Irvin, Irvine, CA, United States

**Keywords:** glioma, platelet-activating factor (PAF), lipid mediators, oncology, stroke

## Abstract

Glioblastoma multiforme (GBM) is an aggressive, highly proliferative, invasive brain tumor with a poor prognosis and low survival rate. The current standard of care for GBM is chemotherapy combined with radiation following surgical intervention, altogether with limited efficacy, since survival averages 18 months. Improvement in treatment outcomes for patients with GBM requires a multifaceted approach due to the dysregulation of numerous signaling pathways. Recently emerging therapies to precisely modulate tumor angiogenesis, inflammation, and oxidative stress are gaining attention as potential options to combat GBM. Using a mouse model of GBM, this study aims to investigate Avastin (suppressor of vascular endothelial growth factor and anti-angiogenetic treatment), LAU-0901 (a platelet-activating factor receptor antagonist that blocks pro-inflammatory signaling), Elovanoid; ELV, a novel pro-homeostatic lipid mediator that protects neural cell integrity and their combination as an alternative treatment for GBM. Female athymic nude mice were anesthetized with ketamine/xylazine, and luciferase-modified U87MG tumor cells were stereotactically injected into the right striatum. On post-implantation day 13, mice received one of the following: LAU-0901, ELV, Avastin, and all three compounds in combination. Bioluminescent imaging (BLI) was performed on days 13, 20, and 30 post-implantation. Mice were perfused for *ex vivo* MRI on day 30. Bioluminescent intracranial tumor growth percentage was reduced by treatments with LAU-0901 (43%), Avastin (77%), or ELV (86%), individually, by day 30 compared to saline treatment. In combination, LAU-0901/Avastin, ELV/LAU-0901, or ELV/Avastin had a synergistic effect in decreasing tumor growth by 72, 92, and 96%, respectively. Additionally, tumor reduction was confirmed by MRI on day 30, which shows a decrease in tumor volume by treatments with LAU-0901 (37%), Avastin (67%), or ELV (81.5%), individually, by day 30 compared to saline treatment. In combination, LAU-0901/Avastin, ELV/LAU-0901, or ELV/Avastin had a synergistic effect in decreasing tumor growth by 69, 78.7, and 88.6%, respectively. We concluded that LAU-0901 and ELV combined with Avastin exert a better inhibitive effect in GBM progression than monotherapy. To our knowledge, this is the first study that demonstrates the efficacy of these novel therapeutic regimens in a model of GBM and may provide the basis for future therapeutics in GBM patients.

## Introduction

**FIGURE 1:**
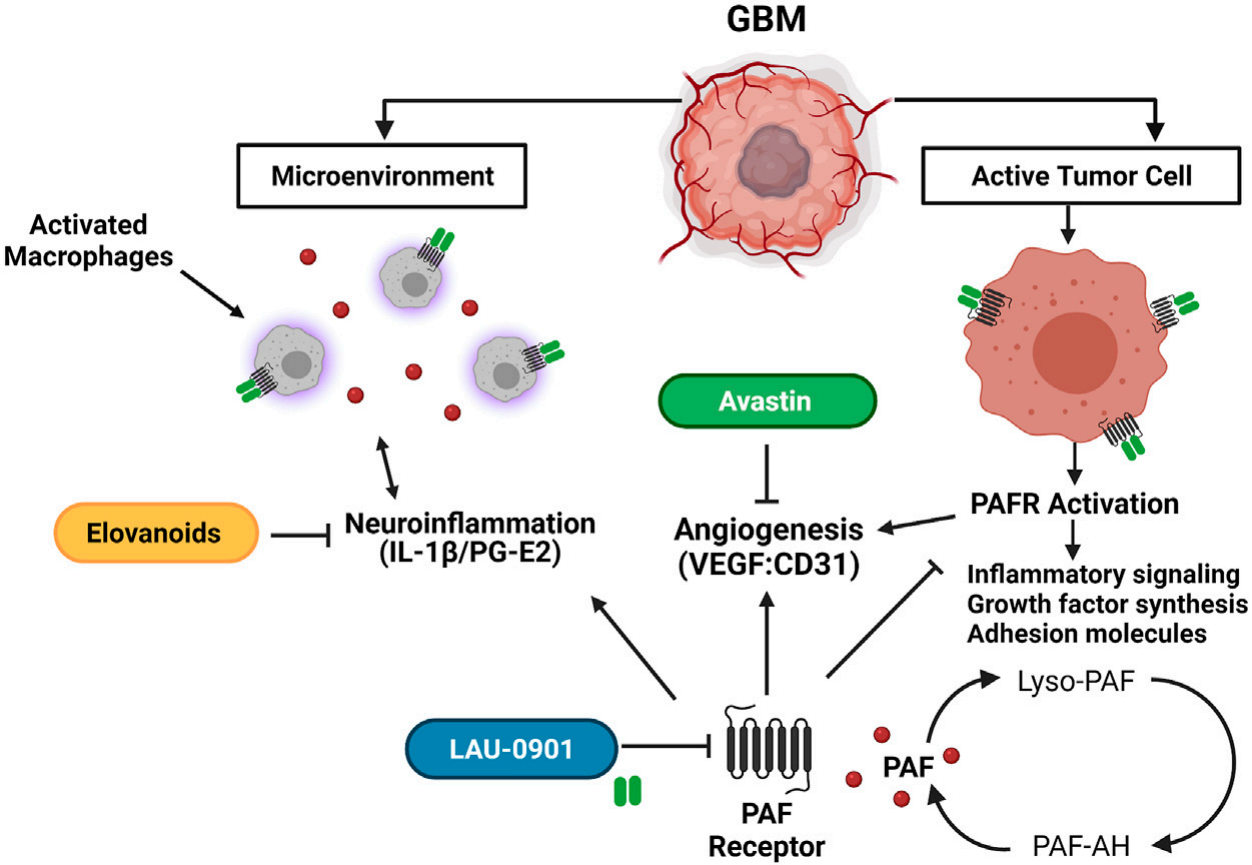
Schematic representation of the potential targets of LAU-0901, Elovanoids, and Avastin in the GBM tumor microenvironment. LAU-0901 a selective PAFR antagonist prevents over-activation of PAFR. Excessive production of PAF and over activation of PAFR increases synthesis of growth factors, adhesion molecules, inflammatory signalin and promotes angiogenesis. ELVs target pro-inflammatory signaling pathways which plays a role in the tumor microenvironment inhibiting proliferation and migration of cancer cells. Avastin is a monoclonal antibody, which prevents VEGF binding, thus inhibiting angiogenesis.

Glioblastoma multiforme (GBM) is a high-grade tumor from glial cells of the central nervous system (CNS), accounting for 49% of malignant brain tumors (Chen et al., 2019; Ostrom et al., 2020). The current standard of care for GBM involves maximal safe surgical resection, radiation, and adjuvant chemotherapy. This conventional approach has shown little impact on the survival and prognosis for patients with GBM due to the heterogeneous, highly proliferative, and invasive nature of GBM (Stupp et al., 2005; Soda et al., 2013; von Neubeck et al., 2015; Mooney et al., 2019). Other strategies that aim to inhibit tumor angiogenesis lead to an adaptive tumor response, transitioning to a more invasive phenotype (Bergers and Hanahan, 2008). Therefore, an elusive goal in brain cancer therapy is to develop targeted approaches against tumorigenic pathways that can effectively lead to long-term, positive outcomes (Woodworth et al., 2014). Recently, emerging mediators to modulate tumor angiogenesis (Avastin), inflammation (LAU-0901), and oxidative stress (Elovanoids) are gaining attention as potential alternatives to combat GBM (Figure 1).

Avastin is a monoclonal antibody against vascular endothelial growth factor (VEGF) approved in 2008 to treat GBM. It has shown a radiographic response rate of up to 40% as a single agent or combined with chemotherapy for GBM recurrence (Mukherji, 2010). However, Avastin has limited efficacy, likely due to adaptive mutations in GBM (Bergers and Hanahan, 2008), leading to no improvement in overall survival compared to standard of care plus radiation in GBM patients (Mukherji, 2010; Ozdemir-Kaynak et al., 2018). Given the inefficacy of available therapeutics for GBM and its high incidence of recurrence, there is a critical need to develop therapies with a higher success rate.

Antagonizing platelet-activating factor (PAF) may be a rational, multipronged therapy for GBM. PAF is a potent pro-inflammatory lipid mediator that has been implicated in the development of cancer and other inflammatory conditions. It is synthesized in circulating and cancer cells and secreted into the tumor microenvironment. PAF has been shown to enhance the production of growth factors, adhesion molecules, and cytokines that have been shown to play a role in tumor angiogenesis and metastasis (Tsoupras et al., 2009; Lordan et al., 2019). Thus, inhibition of PAF biosynthesis may provide an indirect approach to mitigating metastatic angiogenesis of tumors. LAU-0901 (2,4,6-trimethyl-1, 4-dihydro-pyridine-3, 5-dicarboxylic acid) is a highly selective PAF receptor (PAFR) antagonist and a potent inhibitor of apoptosis and inflammatory responses (Bazan et al., 1994; Bazan, 2003; He and Bazan, 2006; Musto et al., 2016; Belayev et al., 2020). It is highly protective when used as an anti-inflammatory in various models (Esquenazi et al., 2004, 2009; He and Bazan, 2006). It has also been shown to have neuroprotective bioactivity when applied to a model of ischemia-reperfusion injury in rats and mice (Belayev et al., 2008, Belayev et al., 2009, Belayev et al., 2012, Belayev et al., 2020).

In addition to anti-inflammatories, mediators of oxidative stress have been closely linked to GBM (Conti et al., 2010). Molecular connections between inflammation, oxidative stress pathways, and the development of gliomas have been established (Alghamri et al., 2021). The tumor microenvironment, which is primarily orchestrated by inflammatory molecules, promotes the proliferation, survival, and migration of such tumors. Recently, we characterized a novel class of lipid mediators termed Elovanoids (ELVs; ELV-N32 and ELV-N34), which are dihydroxylated derivatives of 32:6n3 and 34:6n3, respectively (Bhattacharjee et al., 2017). ELVs are stereoselective mediators made on-demand and derived from very long-chain polyunsaturated fatty acids (VLC-PUFAs) (Calandria et al., 2015). They are a novel class of endogenous pro-homeostatic lipid mediators that protect against excitotoxicity and cell damage and modulate inflammatory responses (Bhattacharjee et al., 2017; Bazan, 2018). Recently, we demonstrated that ELV-N34:6 resulted in reduced infarct volumes, promoted cell survival, and diminished neurovascular unit disruption when administered after experimental focal cerebral ischemia (Bhattacharjee et al., 2017). We predict that ELVs could have a protective effect on neural environments under metabolic catastrophe caused by GBM.

This study aims to investigate the effect of LAU-0901, ELV-N34:6, and Avastin individually and all three compounds in combination to mitigate GBM. Our treatments were compared relative to individual administration of Avastin, which is the latest approved medication to treat GBM. To our knowledge, this is the first study to compare the efficacy of these promising, novel therapies in an orthotopic model of GBM. We hypothesize that the combinatorial application of these agents will potentially improve survival and limit tumor growth in an orthotopic model of GBM. Due to the complex interplay of multiple tumorigenic cascades involved in the dynamics of GBM progression and invasiveness, a combinatorial approach with the treatments investigated in this study may shed light on improving therapy and prognosis of GBM patients.

## Materials and Methods

### Animals and Ethics Statement

Studies were performed according to the National Institutes of Health guidelines and under nationally accepted principles in the care and use of experimental animals. The Institutional Animal Care and Use Committee (IACUC) at the Louisiana State University Health Sciences Center, New Orleans, approved the animal protocols used in this study. Athymic nude female mice (Charles Rivers Laboratories), 6–8 weeks of age, were used in all experiments. Water and food were available for ad libitum consumption. All efforts were made to minimize pain and suffering and reduce the number of mice used in these experiments.

### U87MG Cell Line With Luciferase Reporter

**FIGURE 2:**
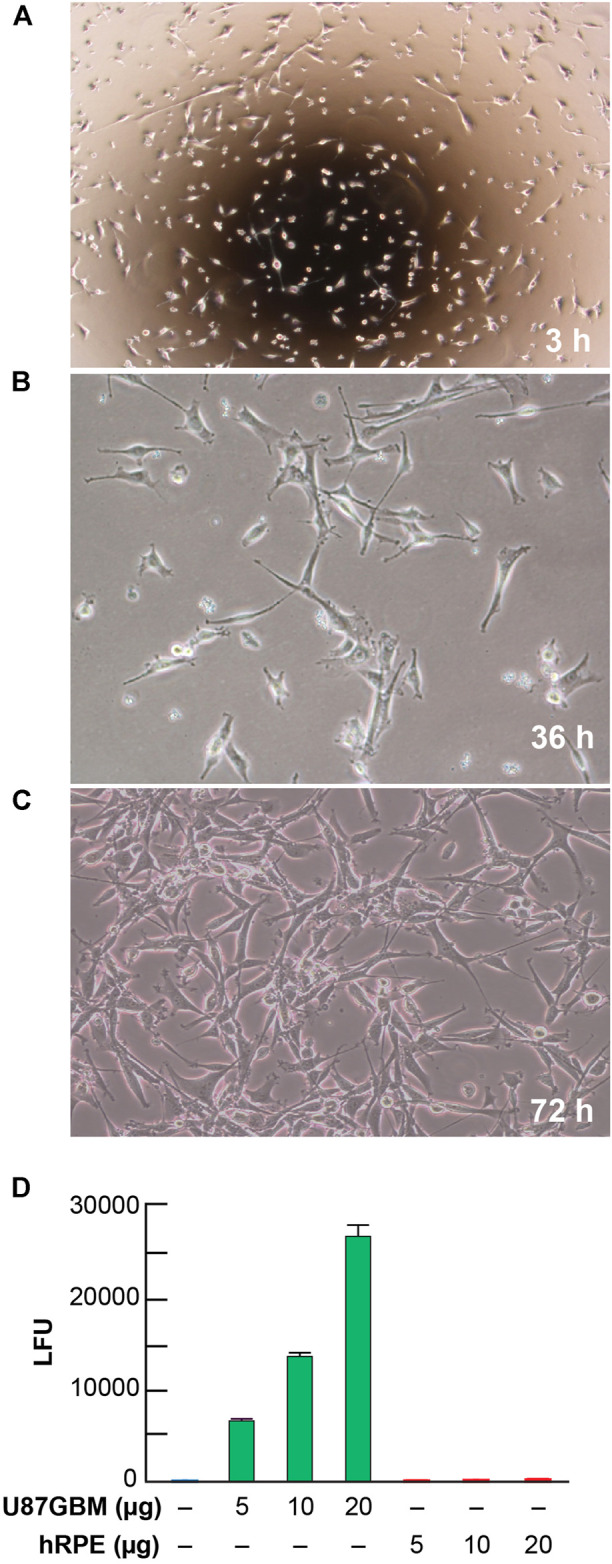
Morphological growth and luciferase activity in U87MG cells. Representative images of U87MG cells at 3 **(A)**, 36 **(B)**, and 72 **(C)** hours at 20x magnification after taking out of Cryofrizide. A luciferase receptor gene was used to tag the U87MG cells. Steady growth and attenuation of the morphological pattern of U87MG cells present at 36 and 72 h. **(D)** Detection of luciferase activity expressed in luciferase units (LFU) in U87MG and hRPE cells. Results are the average of three independent experiments.

The human cell line U87 MG-Red-Flug (U87MG) containing a luciferase-expressing gene was purchased from PerkinElmer (Waltham, MA). Immediately after arrival, cells were stored in liquid nitrogen at the vapor phase until ready to use. Cells were thawed and placed into T-25 mm flasks with Eagle’s MEM (ATCC Cat. No. 30-2003, Manassas, VA) containing 10% FBS (Hyclone, GE Health Care/Fisher Scientific Cat. No. SH300071, Waltham, MA) and puromycin (2 μg/ml). Cells were allowed to grow for up to 72 h at 37°C before sub-culturing them in the same medium. Cell growth was monitored and photomicrographed at 3, 36, and 72 h (Figures 2A–C). 500,000 U87MG and human retinal pigment epithelial (hRPE) cells were allowed to grow separately in six-well plates for 72 h at 37°C, to 80% confluency, in three separate experiments. Cell extracts were made and protein content was adjusted in µg/µL by the Bio-Rad method. Luciferase activity was measured in luciferase units (LFU) using a Glomax 20/20 luminometer in 5–20 µg protein extracts using Luciferin as substrate (Figure 2D). hRPE cells were used in these experiments as controls to show the specificity of luciferase gene expression in the U87MG line.

### Orthotopic Model of GBM

**FIGURE 3:**
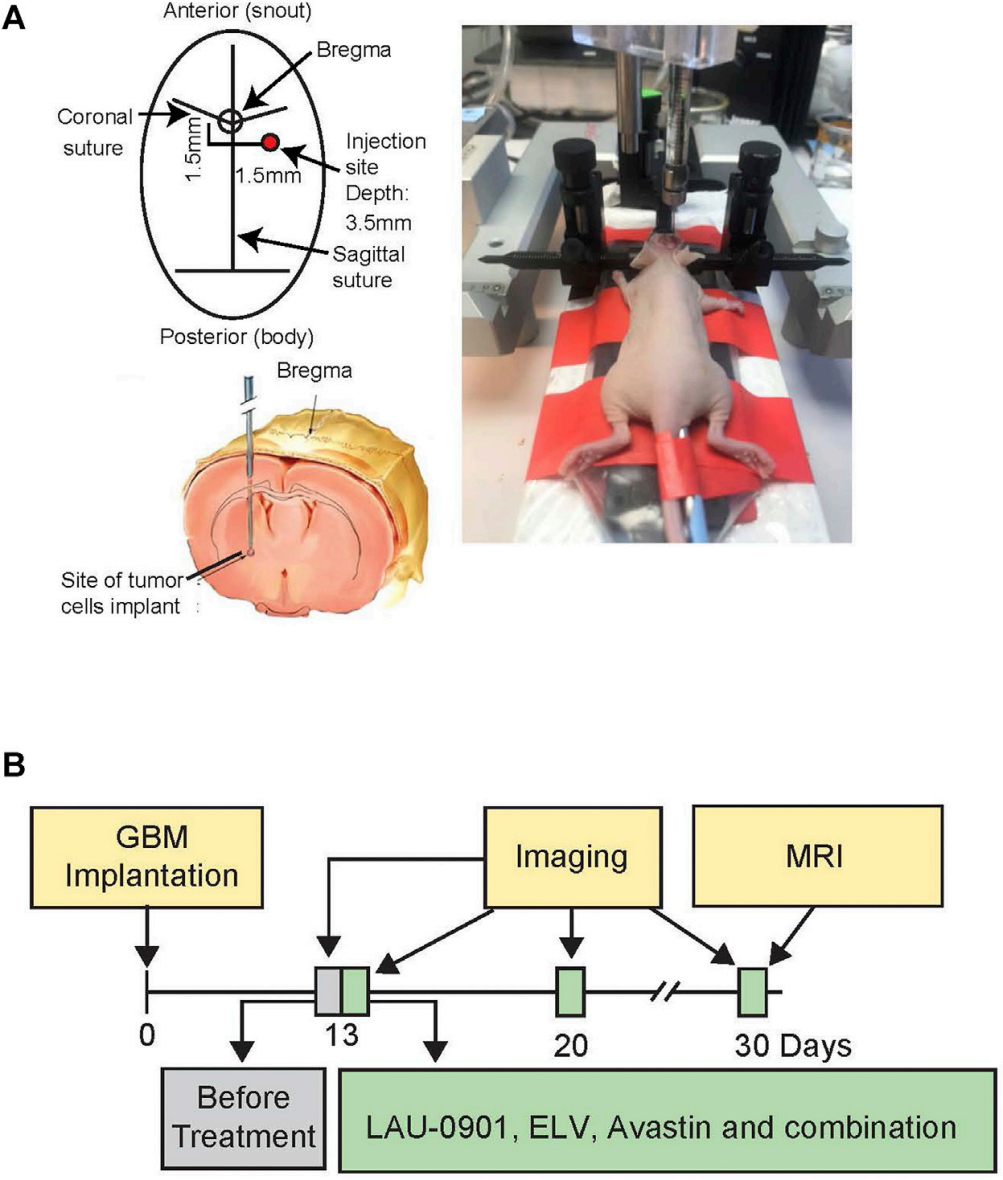
**(A)** Experimental design, showing bregma level and site of tumor cell implantation in anesthetized athymic nude female mice, secured in a stereotactic head frame. **(B)** Timeline showing GBM implantation, imaging, and treatments. Mice underwent stereotactic implantation of the luciferase-modified U87MG cells on day 0 and were monitored during a 30-day survival period. Treatment was started on day 13 post-implantation. *In vivo* bioluminescent imaging was performed on days 13, 20, and 30 post-implantation. On day 30, mice were sacrificed, and *ex vivo* MRI was conducted on perfused brains.

Mice were anesthetized with a ketamine/xylazine cocktail solution (100 mg/kg; 10 mg/kg) and secured in a stereotactic head frame. A midline, 1 cm incision was made over the scalp. Natural tear lubricant was applied to the eyes. For each mouse, 5 × 10^6^ U87MG cells in 5 µL serum-free Dulbecco’s Modified Eagle Medium (DMEM) were injected into the right hippocampus using a 10 µL Hamilton syringe at the following coordinates related to the bregma: 1.5 mm lateral, 1.5 mm posterior, and 3.5 mm in depth. The needle was lowered to 3.5 mm and retracted by 1 mm, before injection (Figure 3A) (Marrero et al., 2014). Instruments to control rectal (CMA/150 Temperature Controller, CMA/Microdialysis AB, Stockholm, Sweden) and cranial (temporalis muscle; Omega Engineering, Stamford, CT) temperatures were closely maintained at 36–37°C before, during, and after the procedure. The incision was sutured using sterile black monofilament nylon 5.0, and the area was cleaned with betadine. Mice were individually caged, observed daily for body weight, temperature, and locomotor changes. Animals were perfused at the end of the 30-day survival and brains removed for *ex vivo* MRI. The experimental design is presented in Figure 3B.

### Treatments

Specific doses of LAU-0901 (30 mg/kg) and ELV 34:6 (5 µg), which have been shown to provide the best neuroprotection in the stroke model (Belayev et al., 2008; Bhattacharjee et al., 2017), and Avastin (10 mg/kg, 0.2 mg/mouse) in the same murine model of glioblastoma (Pechman et al., 2011) were therefore chosen for this study. Mice were randomly and blindly allocated to eight treatment groups. The following treatments were used (*n* = 5–7 per group): 1) LAU-0901, 30 mg/kg, IP; daily x 5 days; 2) ELV 34:6, 30 µg/mouse, IP; once; 3) Avastin 0.2 mg/mouse, IP, weekly x 2 doses (Pechman et al., 2011); 4) Saline in equal volume (0.2 ml/mouse); 5) LAU-0901 + ELV; 6) LAU-0901 + Avastin; and 7) ELV 34:6 + Avastin. For combinatory treatment, LAU-0901 was administered first, followed by Avastin or ELV 5 min later. For ELV combinatory treatment, ELV was administered first, followed by Avastin 5 min later. Treatment was administered on post-implantation day 13, and the bioluminescent imaging (BLI) time course started.

### Bioluminescence Imaging

*In vivo* intracranial tumor growth was quantified by BLI using a Xenogen IVIS200 biophotonic imager (Caliper) facilitated by the Morphology and Imaging Core of the LSU Health School of Medicine. Mice were randomly assigned to individual treatment groups. For each imaging session, mice were injected intraperitoneally with D-Luciferin (150 mg/kg) 5 min before imaging. Anesthesia was administered by isoflurane-oxygen mix (3%) in an XGI-8 system equipped with a vaporizer and induction chamber. Following induction, mice were moved to the IVIS200 imaging chamber equipped with a 5-position manifold and enough nose cones to simultaneously sustain and image groups of five mice. Tumor growth was measured on days 13, 20, and 30 post-implantation. Images were captured and quantified using Living Image 4.1 software based on equivalent regions of interest (ROI) over the head. Emitted radiance values are reported in photons/second, as previously described (Marrero et al., 2014).

### Magnetic Resonance Imaging and Data Analyses

High-resolution *ex vivo* MRI was performed on brains perfused with PBS and 4% paraformaldehyde (PFA) with 8 mM Gd-DTPA (gadobenate dimeglumine; 529 mg/ml; Henry Schein) on day 30. T1-weighted images (T1WI) were obtained on 11.7T Bruker Advance 8.9 cm horizontal bore instrument equipped with an 89 mm (ID) receiver coil (Bruker Biospin, Billerica, MA). We used the following parameters: TR/TE = 1,000/7 ms, matrix = 128^2^, 25 mm × 0.5 mm slices, FOV = 1.8 cm, NEX = 6. MRI acquisition time was ∼9.5 min with an in-plane resolution of 234 um. Cheshire image processing software (Hayden Image/Processing Group, Waltham, MA) was used to manually outline the whole brain and tumor volumes enhanced by Gd deposition. T1WI data were optimized for signal intensity to enhance tumor visualization. Tumors were identified as hyperintense (T1WI) within the striatum and surrounding tissues. Whole brain and tumor volumes (mm^3^) were extracted and analyzed (Jeffes et al., 2005; Blasiak et al., 2013).

### Statistical Analysis

Data are presented as mean values ±SEM. Analysis of variance (ANOVA) with repeated measures, followed by Bonferroni procedures to correct for multiple comparisons, was used to compare groups. Two-tailed Student’s t-tests were used for two-group comparisons. *p* < 0.05 was considered statistically significant.

## Results

### Animal Physiology

All animals in treatment groups survived apart from two-vehicle-treated animals. An increase in body weight in all groups except for the group individually dosed with LAU-0901 was observed. No significant changes were measured in rectal temperature in mice treated with LAU-0901 + Avastin, Avastin, ELV + LAU-0901, or ELV + Avastin during the 30 days of survival compared to vehicle. Treatment with ELV + Avastin increased body weight most significantly by ∼17% on day 30 compared to all treatment groups. In contrast, animals that received vehicles did not experience a significant increase in body weight during the 30-day survival period.

### Evaluation of U87MG-Luc and Bioluminescence Imaging Assays

**FIGURE 4:**
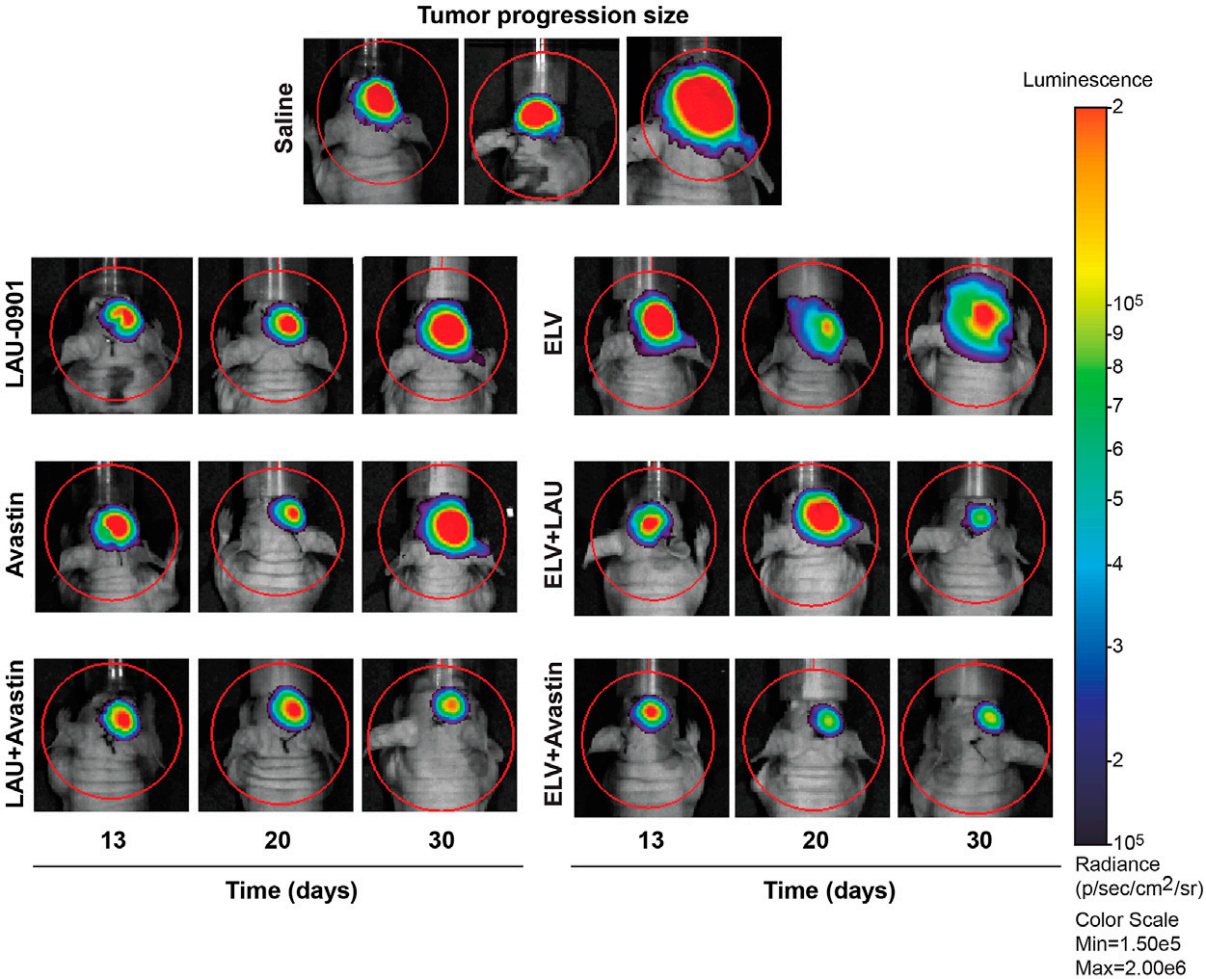
Representative bioluminescent images of the brain tumors from all experimental groups. Mice received treatment on day 13, and tumor growth progression was monitored on days 13, 20, and 30 after implantation. The intensity of light emission is indicated by a colorimetric scale, where red represents the highest amount of light emission, and blue/violet shows the least. There was progressive and rapid tumor growth in the saline group. In contrast, LAU-0901, Avastin, ELV, and combination repress orthotopic GBM.

Representative images of cell cultures showing cell growth measured at 3, 36, and 72 h (Figures 2A–C) revealed steady growth and the morphological pattern of U87MG cells, expressing a luciferase reporter. The level of luciferase activity increased in U87MG cell protein extracts compared to hRPE (Figure 2D). An hRPE cell line was used as a standard control in our assays since they do not contain the luciferase gene. Tumor growth was measured on days 13, 20, and 30 using *in vivo* biophotonic imaging. Representative images of tumor-bearing mice are presented in Figure 4. The emitted radiance correlated with the number of live cells and indicated tumor burden (Figure 4). During the first 13 days, all intracranial tumors increased in size, with a significant difference seen only in ELV and ELV + Avastin treated groups with a *p*-value of *p* = 0.04 and *p* = 0.049 compared to vehicle. There was progressive and rapid tumor growth in the saline group. On day 20, all mice had intracranial tumors, which varied in size, although all treated mice appeared to exhibit smaller tumors than saline-treated mice. On day 30, two mice from the saline group were dead, and the remaining six had extensive tumors.

**FIGURE 5:**
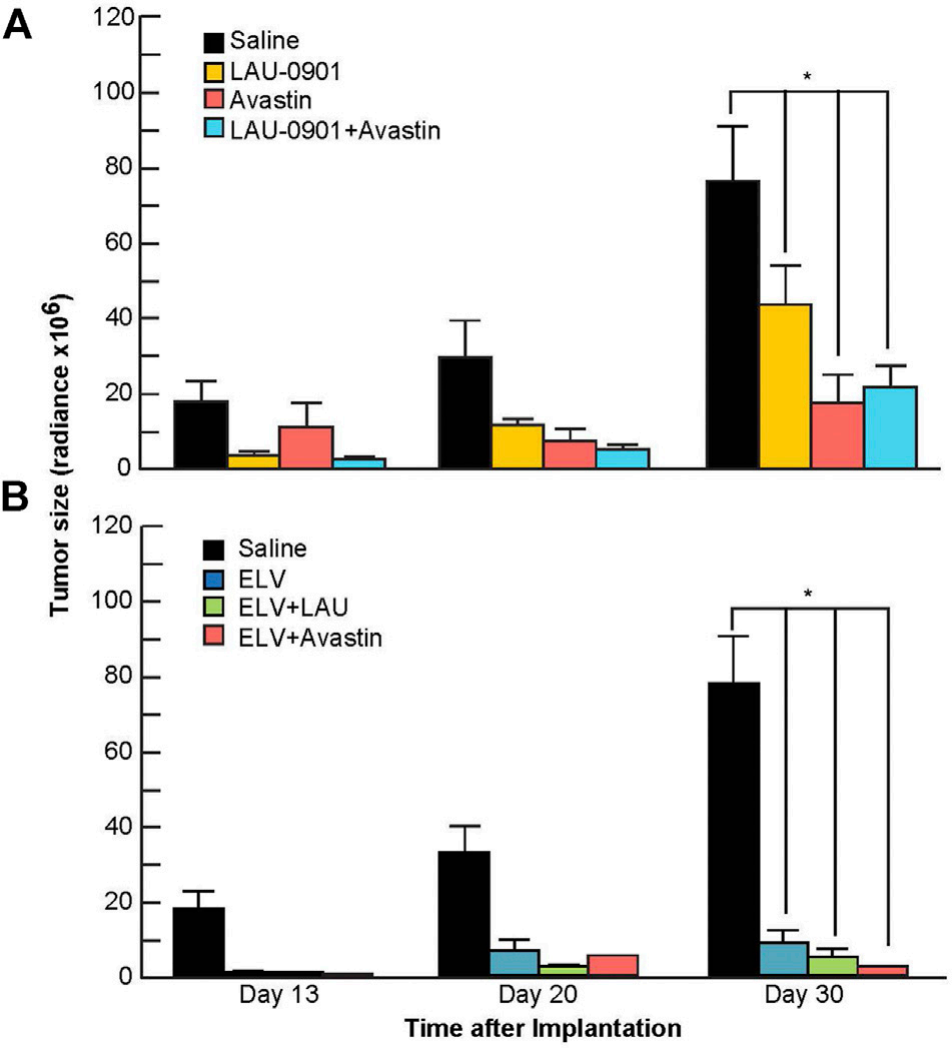
Quantification of bioluminescent signals from tumors. Radiance (Radiance × 10^6^) values from regions of interest in mice from all groups were averaged and compared on days 13, 20, and 30 following intracranial implantation of U87-Luc cells. Tumor-bearing mice treated with **(A)** LAU-0901, Avastin, LAU-0901 + Avastin and **(B)** ELV, ELV + LAU-0901, and ELV + Avastin were observed have significantly reduced tumorigenesis when compared to vehicle-treated mice. All values are mean ± SEM (*n* = 5–7), *p < 0.05.

Quantification of BLI tumor growth over time is presented in Figures 5A,B. Tumor size was reduced by all treatments on day 20 but did not reach statistical significance from the vehicle group. In contrast, tumor size was significantly reduced on day 30 by the following percentages: LAU-0901 by 43%, Avastin by 77%, LAU-0901 + Avastin by 72%, ELV by 86%, ELV + LAU-0901 by 92%, and ELV + Avastin by 96% (Figures 5A,B). Tumor growth was lowest in the ELV + Avastin treatment group and showed the most significant reduction compared to vehicle (Figure 5B).

### T1WI Evaluation of Brain Tumors

**FIGURE 6:**
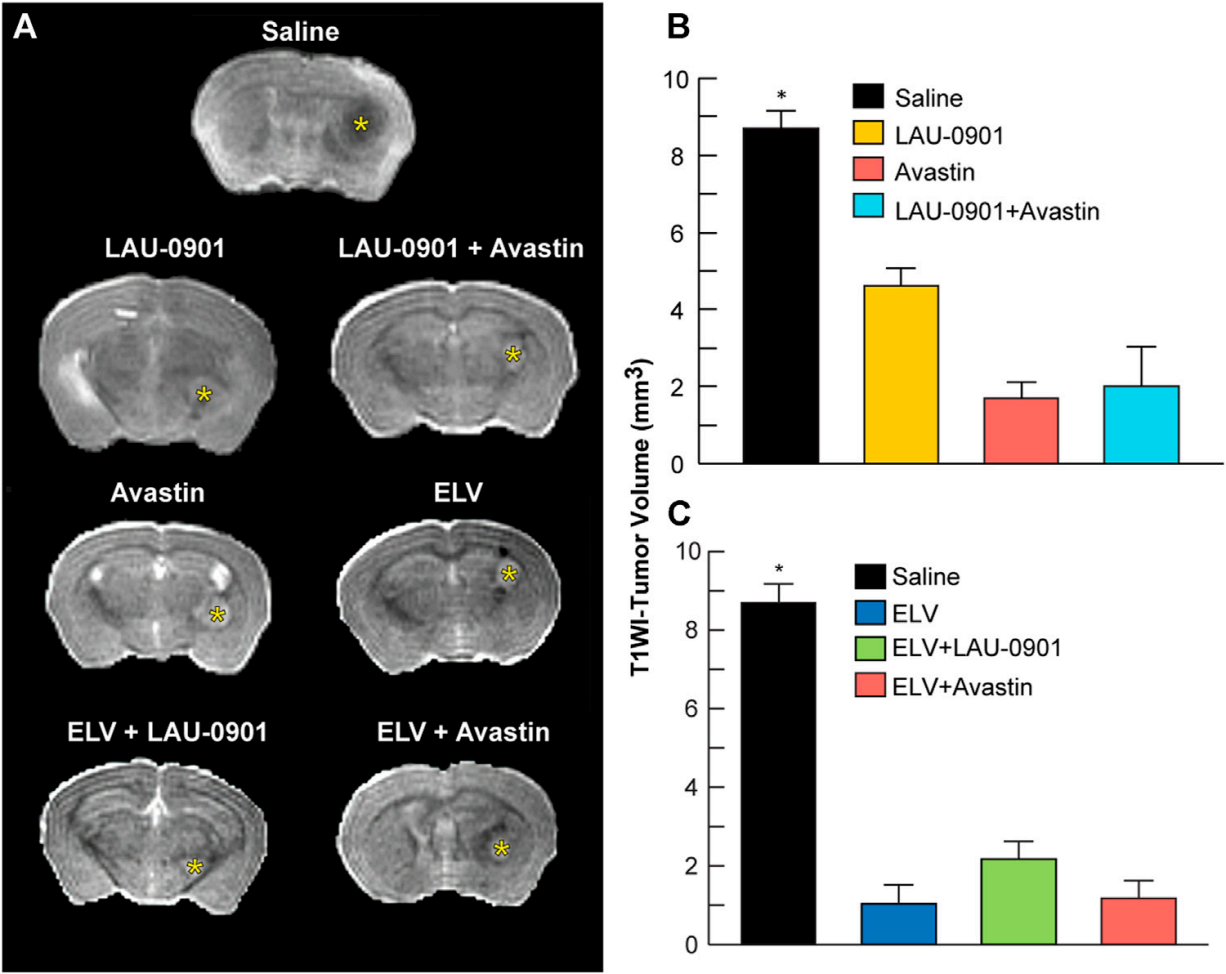
T1WI measurements of tumor volume. **(A)** Representative T1-weighted images from treatment groups. Gadolinium (Gd)-enhanced tumor visualization on T1-weighted MRI (T1WI). *Ex vivo* T1WI of the entire cerebrum was performed to measure brain and tumor volumes. All treatments reduced intracranial tumor growth. Tumor volume was significantly reduced in LAU-0901/Avastin **(B)** and ELV/Avastin **(C)** compared to the vehicle group. All values are mean ± SEM (*n* = 5–7), *p < 0.05.

T1WI revealed more extensive tumor growth in vehicle-treated animals but reduced growth in all animals that received experimental treatments (Figure 6A). Tumor volume was reduced compared to the vehicle by 37% in animals treated with LAU-0901, 67% when treated with Avastin, and 69% when treated with LAU-0901 + Avastin (Figure 6B). Further reduction in tumor volume was observed in groups administered with ELV. We measured an 81.5% reduction when treated with ELV and 78.7% when treated with ELV + LAU-0901. The smallest tumor volume on day 30 was observed in the group treated with ELV + Avastin and showed the most significant (*p* < 0.001) reduction by 88.6% compared to vehicle (Figure 6C).

## Discussion

We have shown here that LAU-0901 (PAF receptor antagonist), ELV (a novel lipid mediator), Avastin (monoclonal antibody against VEGF), and their combination improved survival and reduced tumor volume and growth in the experimental GBM model. Intracranial tumor reduction was confirmed by BLI on days 20 and 30 and by MRI on day 30.

GBM is the most aggressive and lethal malignancy of the CNS, with a poor prognosis and median survival of 8 months (Ostrom et al., 2020). Several treatments have been evaluated in patients with recurrent or progressive GBM without consistent survival benefit (Mooney et al., 2019). One pathologic feature of GBM that distinguishes it from lower-grade glial tumors is the extent of microvascular proliferation. The hypoxic environment of the GBM tumor core influences the sprouting of capillaries from preexisting blood vessels through the upregulation of hypoxia-inducible factor (HIF1-alpha), which triggers the downstream transcription of VEGF. VEGFs activate endothelial cells by binding to VEGF receptor tyrosine kinases to effectively stimulate the endothelial cell proliferation and permeability of vessels to support the metabolic demands of GBM. To that end, Avastin remains the most extensively characterized suppressor of VEGF-A and anti-angiogenetic treatment (Garcia et al., 2020). However, the efficacy of Avastin is limited by adaptive mutations in GBM (Bergers and Hanahan, 2008). As a result, numerous targeted approaches involving Avastin have been investigated, such as its combination with immune checkpoint inhibitors, with encouraging results for treating lung, renal cell, hepatocellular carcinomas, and PARP inhibitor patients with ovarian cancer (Huang et al., 2017; Garcia et al., 2020). Similar to the suppression of tumor growth in the mouse GBM model, the combination of Avastin with other chemotherapeutic agents has been proven effective against non-GBM neoplasm growth (Freitas and Campos, 2019). However, the application of therapies that show improved outcomes in GBM patients remains a challenge. The lack of a durable response is primarily attributed to the acquisition of chemoresistance due to the activation of pathways that enhance cell survival, angiogenesis, and invasion (Woodworth et al., 2014) to treat malignancies such as GBM.

This limitation warrants additional investigation on multipronged approaches to target specific signaling pathways used by GBM to overcome conventional therapies. Therefore, new treatments that can prevent or overcome resistance mechanisms in GBM are needed. So far, no significant efficacy with therapeutic agents alone has been demonstrated (Yamada et al., 2020). Our study intended to investigate the effect of LAU-0901, ELV-N34:6, Avastin and their combination that would significantly increase the probability of survival of mice with intracranial implantation of the luciferase-modified U87MG tumor cells as potential treatments for GBM. This preclinical study shows that individual or concurrent application of LAU-0901, ELV-N34:6, or Avastin can improve survival in the GBM mouse model.

Over-activation of PAFR has been shown to accelerate tumor cell proliferation and other pro-tumorigenic effects (da Silva et al., 2018). Recent studies suggest that PAF receptor-dependent mechanisms are responsible for modifying the tumor microenvironment, including the phenotype of tumor macrophages (da Silva et al., 2017). Excess PAF, which occurs under pathologic conditions, can become neurotoxic, and inhibition of this process enhances neuronal survival (Bazan, 2005; Tian and Bazan, 2005). Thus, PAF represents a rational therapeutic target in GBM. As a novel PAFR antagonist, LAU-0901 has been previously shown to be neuroprotective in inflammation, epilepsy, and ischemic stroke models (Bazan et al., 1994; Bazan, 2005; Belayev et al., 2008, 2009, 2012; Musto et al., 2016). PAF is a potent phospholipid messenger and, when overproduced, acts as an inflammatory mediator that stimulates cell infiltration and expression of cyclooxygenase-2 (COX-2) (Tian and Bazan, 2005; Esquenazi et al., 2009). COX-2 is rapidly induced in response to tissue injury and disease states to mediate events associated with severe inflammatory processes such as lipopolysaccharides, excitotoxicity, cytokines, and growth factors (Qiu et al., 2017). It is upregulated in tumor cells in tissues and accompanied by elevated levels of Prostaglandin E2 and selective COX-2 inhibitors, which have been demonstrated efficacious at reducing proliferation and migration of the U87MG cell line (Qiu et al., 2017). Previously, we demonstrated that LAU-0901 inhibits PAF, which activates the COX-2 pathway when overloaded (He and Bazan, 2006; Belayev et al., 2008, 2012). The significance of COX-2 during pathophysiological conditions presents it as an appealing intervention for inflammatory diseases. Also, PAF accumulation produces CNS damage through intracellular Ca^2+^ overload, reduction in cerebral blood flow (CBF), disruption of the blood-brain barrier, and stimulation of leukocytes (Belayev et al., 2009, 2012). Recently, we established that LAU-0901 increased local CBF when administered 2 h after focal cerebral ischemia (Belayev et al., 2008). We present evidence that the application of LAU-0901 to the GBM model impaired tumor growth, which suggests PAF and COX-2 as potential targets. Our study used BLI and MRI to assess *in vivo* the effects conferred by LAU-0901 alone or in combination with Avastin and ELV in the GBM model. BLI demonstrated that intracranial tumor growth was significantly reduced by treatment with LAU-0901 alone on days 20 and 30. In combination, LAU-0901/Avastin and LAU-0901/ELV had a synergistic effect in decreasing tumor growth by 71–92%. Moreover, tumor reduction was confirmed by MRI on day 30.

ELVs are the first bioactive chemical messengers made from omega-3, a very-long-chain polyunsaturated fatty acid (VLC-PUFAs, n-3) released in response to cell injury or when cells are confronted with adversities for survival (Bhattacharjee et al., 2017; Bazan, 2018). Among the omega-3 family, docosahexaenoic acid (DHA; 22:6n3) is the most abundant PUFA and serves as a precursor of enzymatically derived dihydroxylated derivatives known as docosanoids. These derivatives include potent neuroprotective mediators made “on-demand” when disruptions to homeostasis are impending (Bazan et al., 2011; Serhan et al., 2015). DHA has been shown to reduce the size of tumors and enhance the positive effects of the chemotherapy drug cisplatin while limiting its harmful side effects (Wang et al., 2011). Recent studies have investigated the effect of DHA on GBM cells in cell culture. DHA was shown to exert an anti-tumor effect, and treatment may be responsible for regulating the malignancy of GBM through the esterification of membrane phospholipids, altering permeability and mobility (Ruan et al., 2019). The precursors of ELVs are made by elongation of DHA and catalyzed by ELOVL4 (elongation of very-long-chain fatty acids-4). ELVs counteract oxygen-glucose deprivation, N-methyl-D-aspartate (NMDA)-induced excitotoxicity, or MCAo-induced ischemic stroke (Bhattacharjee et al., 2017; Bazan, 2018). They are rapidly synthesized in the presence of homeostatic disruptors and when cells need to counteract neuroinflammatory responses to protect their integrity and prevent cell death (Bazan, 2018).

Inflammation-induced mutagenesis is a common hallmark of cancer due to the genetic instability it causes. Thus, regulation of inflammatory signaling in the tumor microenvironment may help mitigate the tumors ability to acquire adaptive mutations and resistance to therapy. Mutation rates in inflamed microenvironments have been shown to increase compared to normal tissue (Colotta et al., 2009; Grivennikov et al., 2010). Through modulation of inflammatory signaling by both LAU-0901 and ELV, they may contribute to reducing adaptive mutagenesis, preventing tumor cells from acquiring resistance to therapeutics, thus providing a possible mechanism of LAU-0901 and ELV bioactivity in glioblastoma. We discovered that treatment with ELVs in the mouse GBM model, both alone and in combination with LAU-0901 or Avastin, significantly reduced tumor growth and tumor size by day 30 by 71–92%. Tumor growth was lowest in the ELV + Avastin treatment group and showed the most significant reduction compared to vehicle-treated rats. ELVs greater inhibitory effect than the combination of LAU-0901 + Avastin can be attributed to its potent pro-homeostatic bioactivity targeting multiple signaling pathways. This would contribute to reducing cancer cell proliferation, survival, and migration that results from excessive inflammatory signaling in the microenvironment, inhibiting tumor growth in our model. The use of combination therapy is a centerpiece of cancer therapy. To our knowledge, this is the first study to demonstrate the efficacy of LAU-0901, ELV, and their combination in the experimental GBM model. We found that treatment with our novel therapeutic approach reduced tumor volume and growth following xenograft implantation of human-derived GBM cells. These results provide a basis for further investigation of the use of our novel compounds as potential treatments when applied to a model of GBM. The potentiation of Avastin by LAU-0901 and ELV offers a promising strategy that may ultimately improve clinical outcomes in patients with GBM.

## Data Availability

The original contributions presented in the study are included in the article/supplementary material, further inquiries can be directed to the corresponding author.
